# The effect of facial occlusion on facial impressions of trustworthiness and dominance

**DOI:** 10.3758/s13421-022-01316-z

**Published:** 2022-05-02

**Authors:** Manuel Oliveira, Teresa Garcia-Marques

**Affiliations:** 1 Department of Social, Health, and Organisational Psychology, Utrecht University, Heidelberglaan 1, 3584 CS Utrecht, The Netherlands; 2 William James Center for Research, ISPA – Instituto Universitário, Rua Jardim do Tabaco n°34, 1149-041 Lisboa, Portugal

**Keywords:** Dominance, Face masks, Face perception, Impression formation, Trustworthiness

## Abstract

Recognizing the role that facial appearance plays in guiding social interactions, here we investigated how occlusions of the bottom-face region affect facial impressions of trustworthiness and dominance. Previous studies suggesting that different facial features impact inferences on these traits sustain the hypothesis that wearing a face mask will differently affect each trait inference. And specifically, that trustworthiness impressions will be more disrupted by this type of face occlusion than dominance impressions. In two studies, we addressed this possibility by occluding the bottom face region of faces that were previously shown to convey different levels of dominance and trustworthiness, and tested differences in the ability to discriminate between these trait levels across occlusion conditions. In Study 1 faces were occluded by a mask, and in Study 2 by a square image. In both studies, results showed that although facial occlusions generally reduced participants’ confidence on their trait judgments, the ability to discriminate facial trustworthiness was more strongly affected than the ability to discriminate facial dominance. Practical and theoretical implications of these findings are discussed.

The recent pandemic led to a remarkable transformation of our social lives. With the goal of minimizing viral spread, governments worldwide began to implement and enforce measures such as social distancing, self-isolation, and the use of face masks. In particular, the growing use of face masks highlighted the gaps in our current knowledge of social face perception. In social and professional contexts where the use of face masks is (or became) more prevalent, people cannot rely on the same amount of facial information to form impressions about each other. Yet, the degree to which occlusions of face regions (e.g., by a mask) distort social judgments has remained largely unexamined. In this paper, we present one study documenting the implications of wearing a face mask on facial impressions of personality. Below we underline the empirical and theoretical relevance of these data to our current knowledge on how people subjectively form impressions of personality based on facial appearance.

Regardless of accuracy, people regularly rely on facial appearance to guide their social interactions. A fleeting look at a face allows us to identify a person, classify a person into multiple social categories (e.g., gender, age, ethnicity; Bodenhausen & Macrae, 2006; Mason et al., 2006; Zebrowitz, 2006), or learn about someone’s emotional state (Darwin, 1872; Ekman & Oster, 1979; Horstmann, 2003). Facial information is automatically processed, and short exposures to faces as fleeting as 100 ms (or less) are sufficient to form an impression about someone on a variety of social traits (Bar et al., 2006; Todorov et al., 2009; Willis & Todorov, 2006). And importantly, research has shown that most trait inferences from faces ultimately reflect perceptions of someone’s trustworthiness and dominance (Oosterhof & Todorov, 2008; see also Lin et al., 2020, and Sutherland et al., 2013, who found similar and additional relevant dimensions).

The widespread use of face masks introduced a new challenge to our current knowledge on facial impressions. The current literature does not yet, however, offer direct empirical evidence clarifying the effects of wearing a face mask on facial impressions of trustworthiness and dominance. This is, however, a question holding both practical and theoretical relevance. Selective facial occlusions may interfere differently with impression formation processes. Examining whether and how this happens clarifies the relevance of different facial regions to these processes.

Although there are studies showing the impact of different types of facial occlusion on facial impressions of personality (e.g., Bartolini et al., 1988; Graham & Ritchie, 2019; Hellström & Tekle, 1994; Leder et al., 2011; Santos & Young, 2011; Terry & Krantz, 1993), fewer studies have focused on identifying the facial features (or combinations thereof) that uniquely predict judgments on both of the central dimensions of trustworthiness or dominance, or on whether and how these judgments are affected by occlusions of the bottom-face region. Given the impact of face occlusions on facial impressions, it is an empirical question whether the concealment of bottom-face features by masks (i) disturbs the general process of trait inferences – by interfering with both trustworthiness and dominance judgments – and/or (ii) interferes more with one dimension relative to the other.

Face masks conceal specific features such as the mouth, nose, chin, and cheekbones. Knowing which of these features overlap with diagnostic features of each judgment allows us to anticipate if and how masks can distort facial impressions. Using a variety of methodological approaches, previous studies have consistently found that dominance and trustworthiness judgments are both informed by similar facial regions, such as the eyes, eyebrows, and hair (Dotsch & Todorov, 2012; Robinson et al., 2014), all of which dispersed across the top- and bottom-face regions. Santos and Young (2011) found that the internal features of a face, enclosing the eyes, nose, and mouth, were critical for facial impressions of trustworthiness. In addition, Vernon et al. (2014) found evidence supporting that the mouth region is likely a major cue to these impressions. Such findings support the prediction that an occlusion of the mouth region would reduce the trustworthiness signal conveyed by a face, thereby impairing the discriminability between high and low trustworthiness.

Importantly, previous studies found that while both the eye and the mouth regions were important for trustworthiness judgments, dominance judgments were mostly supported by the eyebrows, skin saturation, and facial shape features including the delineation of a face, wider chins, and narrower heads (Dotsch & Todorov, 2012; Robinson et al., 2014; Toscano et al., 2014; Vernon et al., 2014; Windhager et al., 2011). These findings converge with evidence presented by Oosterhof and Todorov (2008, p. 11090, Study 10) showing that variations of facial shape were predictive of dominance, but not of trustworthiness judgments. Nevertheless, the literature remains rather mixed regarding an exclusive association of facial shape features to dominance judgments. While some studies found that facial shape features were relevant to trustworthiness impressions (Kleisner et al., 2013; Stirrat & Perrett, 2010), others – more directly focused on the relative contribution of facial features to both judgment dimensions – have found that facial shape was a stronger predictor of dominance impressions compared to trustworthiness impressions, which in turn were more strongly predicted by features resembling the expression of happiness (Jaeger & Jones, 2022).

Altogether, these studies suggest that, although both trustworthiness and dominance judgments are informed by features that remain visible in masked faces (viz., eyes, eyebrows, and hair), facial cues to trustworthiness (i.e., mouth region) are concealed by masking to a larger extent than facial cues to dominance (i.e., facial shape, eyebrows). This suggests the hypothesis that, while both judgments would be similarly disturbed by facial masking, trustworthiness judgments may suffer more distortion relative to dominance judgments.

More recently, studies conducted during the COVID-19 pandemic have already started to shed light on the effects of face masks on trustworthiness and dominance judgments. While examining how face masks impacted social judgments of Black and White faces, Oldmeadow and Koch (2021) found that while masking increased the perceived trustworthiness and even reduced race effects on facial judgments, it did not have an impact on perceived dominance. This increase of perceived facial trustworthiness in mask wearers was also found by Olivera-La Rosa et al. (2020), leading these authors to question whether such an effect could be resulting from an increased desirability of the behavior to wear a mask during a pandemic, fuelled by the internalization of an emergent social norm to wear masks.

## The present research

In two studies, we investigate how the occlusion of the bottom-half of the face affects facial impressions of trustworthiness and dominance with an emphasis on how it impacts the ability to discriminate between high and low facial trustworthiness or dominance. In Study 1, we examined the impact that face masks would have on facial impressions and expected to find that face masks would disrupt impressions of trustworthiness to a greater extent than impressions of dominance. In Study 2, we clarified that the effect was associated with the occlusion of facial features itself, instead of with any other attributes of the object occluding the face. In our approach, we simply occluded facial regions using a (more neutral) digital square image.

In both studies, we relied on the participants’ ability to discriminate target faces that were previously shown to elicit specific judgments of higher or lower trustworthiness or dominance. These target faces were exemplars of two previously validated face stimulus sets extracted from Oh et al. (2020). The first was composed of artificial computer-generated faces (CG faces) whose facial features were manipulated to convey high and low trustworthiness or dominance. The second set reflected identical manipulations but was composed of real-life faces originally obtained from the Face Research Lab London Set (DeBruine & Jones, 2017). These materials ensure high experimental control over the dominance and trustworthiness signal conveyed by stimuli and take into account the interaction between the gender of the stimulus and of the perceiver. The two sets are highly complementary, as they differ both in the degree of experimental control over stimulus features and their ecological validity. CG faces are bald, look artificial, and exhibit higher variation in gender and skin reflectance within each dimension, thus maximizing the trait signal for each dimension, whereas real-life faces vary in hairstyle and reflect natural face variability. Using these materials, we tested the impact of face masks on how participants’ trait judgments discriminate between faces that reflect opposite poles of either dominance or trustworthiness.

In general, we expected to find evidence supporting the notion that facial occlusions of the bottom-face region (by masks or other objects) would interfere with the trait inference process involved in facial impressions. If any differences were to be found between the impact of facial occlusion on each judgment dimension, we assumed that facial occlusions would be less detrimental to the perception of dominance. Specifically, we expected that if facial occlusion of the bottom-face region impairs the discriminability between faces conveying opposite poles of a dimension, this would be clearer for faces varying in trustworthiness.

## Study 1

### Method

#### Participants

Seventy people (50% female, 50% male, *M*_age_ = 27.33, *SD*_age_ = 5.62, age range = 18–40 years) were recruited via Prolific Academic to participate in the study in exchange for £1.25. Our sample included native English speakers living in different countries at the time of data collection (Australia: 15.71%, Greece: 1.43%, Ireland: 5.71%, UK: 41.43%, USA: 5.71%, Unknown: 30%). All participants were included in the analyses. Each participant participated in both Study 1a and Study 1b. We obtained informed consent from all participants.

#### Context of data collection

Data collection took place at the end of May 2020, amidst the COVID-19 worldwide pandemic. During this period, many countries implemented public health measures to minimize contagion. There was considerable variability in how and when different countries implemented them, but the most common included an order to stay at home as much as possible, a strong recommendation to wear surgical face masks in public spaces, and/or to keep a distance of around 1.5 m from other people. Most of our participants were residing in the UK at the moment of data collection, which coincided with the period of the first national lockdown in this country (House of Commons Library, 2021).

#### Design

Participants were randomly assigned to the between-participants conditions of our design, which aimed to counterbalance the materials’ features and the positioning of judgment scales on the screen (i.e., trustworthiness scale above or below dominance scale). The study followed a within-participants design defined by 2 (Masking: masked vs. unmasked) × 2 (Judgment: dominance vs. trustworthiness) × 2 (Face Dimension Pole: high vs. low), with judgment ratings as the dependent variable. The positioning of judgment scales on the screen (i.e., trustworthiness above or under dominance) was counterbalanced between participants. Face stimulus features such as manipulated trait dimension, dimension pole, facial gender, gender of face model producers, and face identity were counterbalanced within participants.

#### Power considerations

Sample size was determined before any data analysis. A priori power simulations were conducted using R package Superpower v0.0.3 (Lakens & Caldwell, 2019). Based on effect sizes obtained for similar facial trait judgments in previous studies (Dotsch & Todorov, 2012), we simulated our theorized pattern of results for each three-way interaction in each within-subjects GLM involving each judgment dimension. Results suggested 46 as the optimal sample size to detect with 80% power the smallest of the simulated effect sizes (*η*_p_^2^_trustw. judgments_ = 0.42, *η*_p_^2^_dominance judgments_ = 0.16), assuming α = .05, a correlation among repeated measures of .30, and a false discovery rate correction (Benjamini & Hochberg, 1995) for multiple comparisons. Sample size was increased to 70 on the basis of available financial resources, which increased power to 100% and 89% for effects involving trustworthiness and dominance judgments, respectively (simulation report available at: https://osf.io/4xvem/).

#### Stimuli

**Fig. 1:**
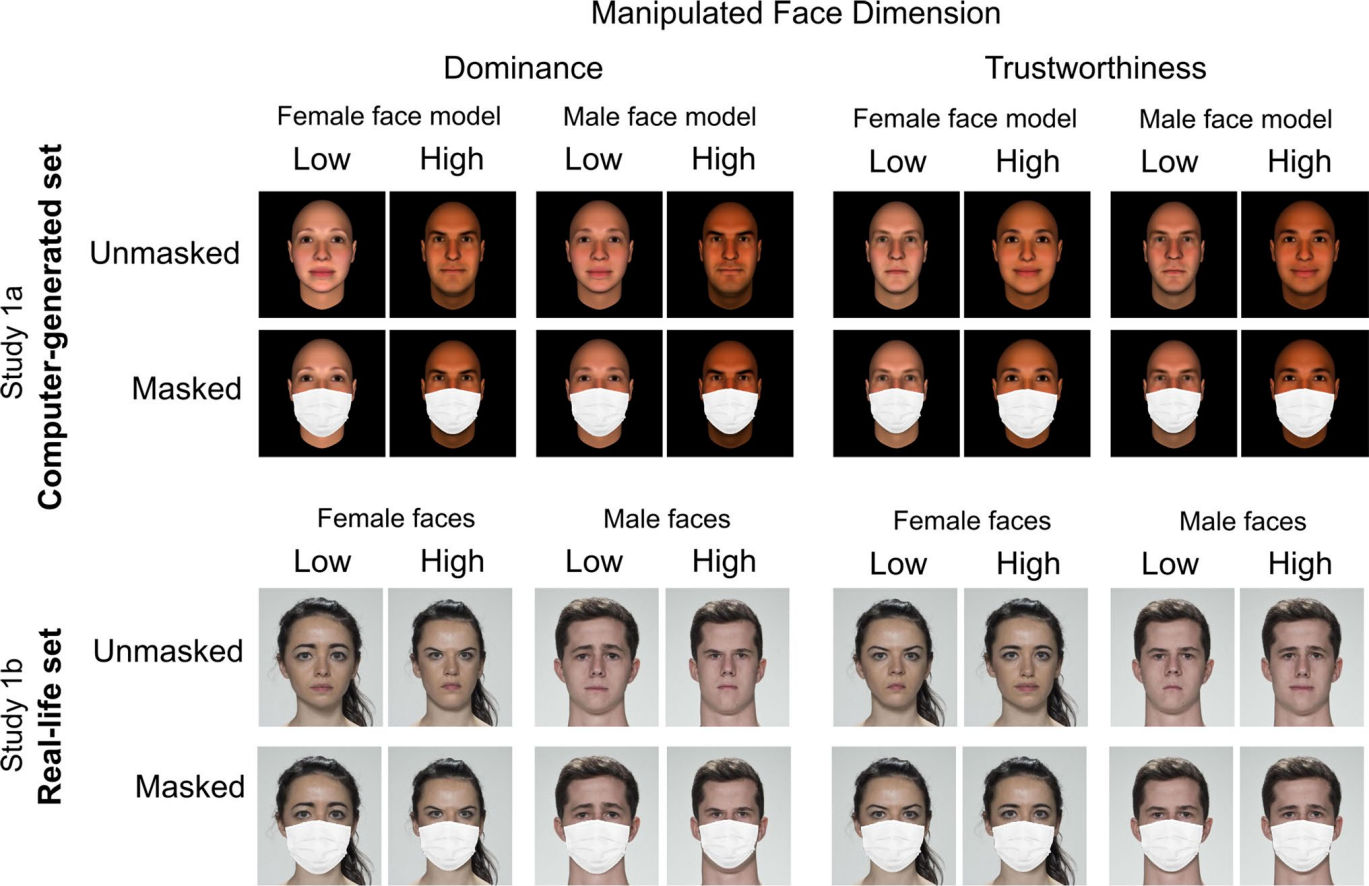
Exemplars of the computer-generated (CG) and real-life face sets used in Studies 1a and 1b, respectively. Unmasked stimuli are extracted from the materials generated by Oh et al. (2020). For CG faces, the female and male face models refer to the gender of the average-face used to generate the exemplars (see Oh et al., 2020). For the sake of simplicity, this figure ignores the perceiver model exemplars within each face gender model

Two sets of face stimuli were used (Study 1a and Study 1b). The set used in Study 1a consisted of computer-generated (CG) face images. The set used in Study 1b consisted of real-life face photographs. Both were obtained from the materials generated by Oh et al. (2020). These faces were generated by computer models of facial trustworthiness and dominance for both male and female faces. In addition, each face gender model of trustworthiness and dominance had two versions: one generated with female perceivers, and another with male perceivers. Facial dimension model, face gender model, and gender of model’s producers were counterbalanced in each face set. For the current study, we used only the extreme levels of each facial dimension model reflecting the low (-3 *SD*) and high pole (+3 *SD*) on a normally distributed dimension (see Fig. 1 for examples). A masked version was then created for every face, thereby doubling the total number of stimuli in each set.

##### Study1a: Computer-generated faces

Thirty-two faces of four different identities were randomly sampled from the CG face set generated by Oh et al. (2020, Study 1a). Unlike real-life faces, the two extremes of each face model differed sufficiently to act as distinct identities. For this reason, the number of identities in the CG set is lower than in the real-life set. Another difference between the sets is that CG faces vary in skin tone and reflectance (see also Todorov et al., 2013a).

##### Study 1b: Real-life faces

Thirty-two faces of eight different identities (four male, four female, *M*_age_ =26.3 years, *SD*_age_ = 5.95 years) of White ethnicity were randomly sampled from the real-life face set generated by Oh et al. (2020, Study 1b), originally extracted from the Face Research Lab London Set (DeBruine & Jones, 2017). Unlike CG faces, the identity of real-life faces reflecting opposite extremes of the same dimension is well preserved. Thus, to prevent repeated exposure to the same identity, we created different subsets where no two extremes of the same model dimension occurred, while equally counterbalancing the factors described above. Individuals depicted in real-life photographs provided consent for their images to be "used in lab-based and web-based studies in their original or altered forms and to illustrate research (e.g., in scientific journals, news media or presentations)" (DeBruine & Jones, 2017; see https://figshare.com/articles/Face_Research_Lab_London_Set/5047666). The subset of face stimuli used in the current study can be found online at: https://osf.io/4xvem/

#### Facial masking

**Fig. 2:**
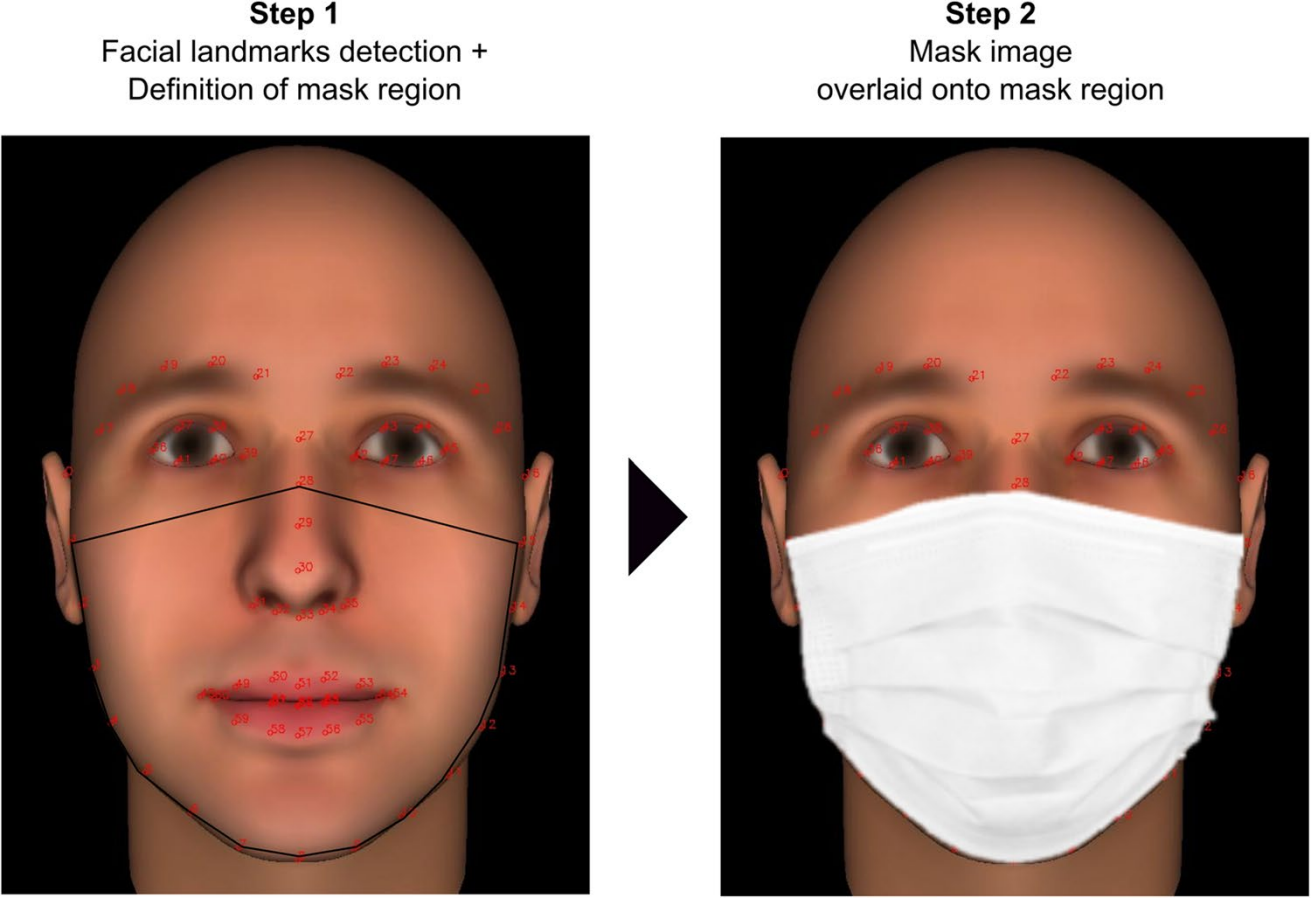
Illustration of the automated facial masking procedure using facial landmark detection

Facial masking was implemented using the OpenCV v3.4.2 and dlib v19.19 modules in a Python 3.7 environment. A masked version of each face image was created by overlaying an edited image of a medical-looking facial mask (retrieved from Google Images) onto each face image. To deal with variation in facial shape, we developed a program that automatically and dynamically resizes and fits the mask image to a fixed configuration of facial landmarks defining the face region typically covered by a face mask (see Fig. 2).

#### Procedure

The experiment was run using Qualtrics software. After consenting to participate in the study, participants were informed that the goal of the study was to investigate how people form personality impressions based on facial appearance when a face is either fully visible or partially covered by a facial mask. Instructions emphasized that: the focus was on first impressions; there were no correct or wrong answers; and that, when in doubt, participants should respond based on their “gut feeling.” (Full instructions available at: https://osf.io/4xvem/)

Study 1a and Study1b were defined by two blocks presented in a fixed order where Study 1a (CG faces) preceded Study 1b (real-life faces). Both masked and unmasked faces were presented in random order within each block. During each trial, the stimulus was presented at the center of the screen and participants were asked to judge the stimulus on both dominance and trustworthiness using a scale ranging from 1 (Very untrustworthy/submissive) to 9 (Very trustworthy/dominant). The positioning of the two judgment scales under a face stimulus on the screen of each trial was counterbalanced between participants, such that the trustworthiness scale was displayed above the dominance scale or vice versa. After completing the task, participants provided demographical information and responded to several exploratory questions. These questions inquired about familiarity with face masks, and several aspects of task difficulty such as easiness of using each judgment rating scale, easiness of judging each judgment on a masked face, and easiness of judging a Real-life or a CG face (for more details see materials deposited in OSF repository). All these additional questions were rated on a 9-point rating scale ranging from 1 (Very unfamiliar/difficult) to 9 (Very familiar/easy). Next, they were thanked, debriefed, and compensated.

### Results and discussion

For ease of interpretation and strength of replicability, we analyzed computer-generated and real-life stimuli separately (Study 1a and 1b, respectively). We expected to reach the same conclusions regardless of the stimulus set, and we should clarify that any potential differences emerging between them were not expected to qualify the hypothesized effects of masking on judgments. For each stimulus set, we first addressed whether facial masking impaired the discriminability between impressions of faces conveying opposite poles of a dimension (e.g., high vs. low dominance/trustworthiness). We ran separate analyses by stimulus Face Dimension (i.e., faces manipulated on dominance or trustworthiness). We expected (i) that masking would affect each judgment differently; (ii) effect sizes reflecting the difference between target face poles (i.e., discriminability) would be larger for unmasked faces relative to masked faces; and (iii) larger differences between these effect sizes (i.e., unmasked discriminability vs. masked discriminability) for trustworthiness judgments compared to dominance judgments.

#### Inter-rater agreement

Intraclass correlation coefficients (see Shrout & Fleiss, 1979) were calculated to assess inter-rater agreement for dominance and trustworthiness judgments, separately for each level of stimulus masking and by stimulus type. Results are listed in Table 1. Overall agreement was high for both judgments, with dominance judgments showing a slightly higher agreement compared to trustworthiness judgments.

**Table 1:** Inter-rater agreement results for dominance and trustworthiness judgments by type of face occlusion and stimulus type, for Studies 1 and 2

Study 1: Faces occluded by face masks				
Stimulus type	Judgment	Masking	*ICC* (2, 70)	*ICC* 95% CI
CG	Dominance	Masked	.97 ***	[.930, .997]
		Unmasked	.99 ***	[.963, .998]
	Trustw.	Masked	.96 ***	[.888, .995]
		Unmasked	.98 ***	[.959, .998]
Real-life	Dominance	Masked	.98 ***	[.941, .997]
		Unmasked	.98 ***	[.938, .997]
	Trustw.	Masked	.91 ***	[.787, .990]
		Unmasked	.91 ***	[.771, .989]
Study 2: Faces occluded by square image				
Stimulus type	Judgment	Face dimension	*ICC* (2, 70)	*ICC* 95% CI
Real-life	Dominance	Dominance	.98 ***	[.941, .999]
		Trustw.	.98 ***	[.932, .999]
	Trustw.	Dominance	.91 ***	[.679, .999]
		Trustw.	.94 ***	[.790, .999]

*** *p* < .001

*ICC* intraclass correlation coefficient, *CI* confidence interval, *CG* computer-generated

#### Familiarity with face masks

On average, participants reported a somewhat low familiarity with face masks (*M* = 4.51, *SD* = 2.21) on a 9-point scale ranging from Very unfamiliar (1) to Very familiar (9).

#### Difficulty of judging face stimuli

On average, participants reported that it was easier to judge Real-life faces (*M* = 5.23, *SD* = 2.04) compared to CG faces (*M* = 4.06, *SD* = 2.17), *t*(108) = -5.10, *p* < .001, *d* = 0.55; that it was easier to use the dominance rating scale (*M* = 4.94, *SD* = 2.40) compared to the trustworthiness rating scale (*M* = 4.34, *SD* = 2.26), *t*(108) = 2.63, *p* = .009, *d* = 0.26; and, importantly, that it was easier to judge the dominance of masked faces (*M* = 4.23, *SD* = 2.05) comparated to judging the trustworthiness of masked faces (*M* = 3.67, *SD* = 2.20), *t*(108) = -4.79, *p* < .001, *d* = 0.26.

#### Statistical analyses

Analyses were conducted using R 4.1.0 (R Core Team, 2021). All statistical tests reported are two-sided.

### Study 1a: Computer-generated faces

**Fig. 3:**
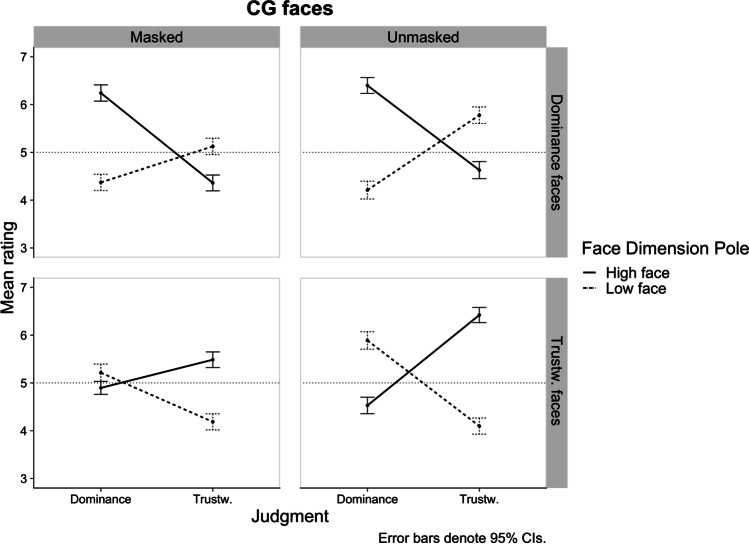
Study 1a plot of the three-way Judgment × Masking × Face Dimension Pole interactions by Face Dimension. Horizontal dotted line represents the middle point of the rating scale

To investigate the impact of facial masking on participants’ ability to discriminate trustworthiness and dominance between the high and low face exemplars of a dimension, we conducted a 2 (Masking: masked vs. unmasked) × 2 (Face Dimension Pole: high vs. low) × 2 (Judgment: dominance vs. trustworthiness) within-participants ANOVA with judgment ratings as the dependent variable, separately by Face Dimension. Figure 3 shows the pattern of the three-way interactions by Face Dimension for computer-generated stimuli. Table 2 lists all the *p*-values and effect sizes for the differences between Face Dimension Pole levels for each condition of the three-way design. A false discovery rate correction (*FDR*; Benjamini & Hochberg, 1995) was applied to *p*-values of multiple comparisons.

**Table 2:** Study 1a: Effect sizes of the difference between the ratings of the high and low poles of a face dimension for each condition

			Face dimension pole			
Face dimension	Judgment	Masking	High *M* (*SD*)	Low *M* (*SD*)	High vs. Low	Cohen’s *d* [95% CI]
Dominance	Dominance	Masked	6.24 (1.48)	4.37 (1.49)	*p* < .001	0.96 [0.76, 1.17]
		Unmasked	6.40 (1.42)	4.21 (1.58)	*p* < .001	1.01 [0.81, 1.21]
	Trustw.	Masked	4.36 (1.40)	5.12 (1.50)	*p* < .001	-0.30 [-0.47, -0.13]
		Unmasked	4.63 (1.57)	5.78 (1.53)	*p* < .001	-0.36 [-0.53, -0.19]
Trustw.	Dominance	Masked	4.90 (1.22)	5.21 (1.52)	*p* = .018	-0.30 [-0.47, -0.13]
		Unmasked	4.53 (1.47)	5.89 (1.55)	*p* < .001	-0.70 [-0.89, -0.52]
	Trustw	Masked	5.49 (1.42)	4.19 (1.38)	*p* < .001	0.73 [0.55, 0.92]
		Unmasked	6.42 (1.44)	4.10 (1.44)	*p* < .001	1.08 [0.88, 1.30]

*n* = 280 for all cell means; positive (negative) Cohen’s *d* values indicate higher mean rating for the high (low) pole of face dimension. A Benjamini-Hochberg correction was applied to all *p*-values in this analysis

#### Trustworthiness faces

As expected, the three-way interaction was significant, *F*(1, 69.01) = 47.06, *p* < .001, *η*_p_^2^ = .405, 95% CI [.233, .546]. The simple interaction effects at each level of Judgment revealed that the Masking × Face Dimension Pole interaction was significant for both judgments and stronger for judgments of Dominance, *F*(1, 69) = 33.30, *p* < .001, *η*_p_^2^ = .326, 95% CI [.156, .477], than for judgments of Trustworthiness, *F*(1, 69) = 30.27, *p* < .001, *η*_p_^2^ = .305, 95% CI [.138, .459]. Follow-up comparisons further clarify that the differentiation between trustworthy and untrustworthy faces in terms of perceived dominance and trustworthiness was statistically significant regardless of masking (see Table 2 and Fig. 3), and that judgments of dominance of faces manipulated on trustworthiness were more strongly impaired by masking than judgments of trustworthiness.

Comparisons between masked and unmasked faces of the same dimension and pole, further revealed that while masked trustworthy faces were significantly perceived as less trustworthy than unmasked trustworthy faces (*p*_FDR_ < .001, *d* = 0.65, 95% CI [0.48, 0.82]), impressions of untrustworthy faces do not significantly differ across masking levels (*p*_FDR_ = .532, *d* = -0.06, 95% CI [-0.23, 0.10]). This suggests that trustworthiness perceptions of high facial trustworthiness were more strongly impaired by masking compared to trustworthiness perceptions of low facial trustworthiness. The same comparisons with dominance judgments reveal that masked trustworthy faces were significantly perceived as less dominant than unmasked trustworthy faces (*p*_FDR_ = .021, *d* = -0.27, 95% CI [-0.44, -0.11]), and that unmasked untrustworthy faces were perceived as more dominant than masked untrustworthy faces (*p*_FDR_ < .001, *d* = 0.44, 95% CI [0.27, 0.61]). This again suggests that judgments of dominance were more strongly impaired by masking than judgments of trustworthiness. Finally, the significant Face Dimension Pole × Judgment interaction, *F*(1, 69) = 138.52, *p* < .001, *η*_p_^2^ = .668, 95% CI [.539, .753], indicates a negative relationship between judgments such that the higher the facial trustworthiness the lower the perceived dominance.

#### Dominance faces

The three-way interaction was significant, *F*(1, 69) = 6.23, *p* = .015, *η*_p_^2^ = .083, 95% CI [.003, .226]. However, the simple interaction effects at each level of Judgment revealed that the Masking × Face Dimension Pole interaction was only significant for trustworthiness judgments, *F*(1, 69) = 5.75, *p* = .019, *η*_p_^2^ = .077, 95% CI [.001, .218] (vs. Dominance judgments: *F*(1, 69) = 2.21, *p* = .141, *η*_p_^2^ = .031, 95% CI [.000, .148]). Crucially, and as expected, this indicates that masking did not impact the ability to discriminate the dominance between dominant and submissive faces. However, while the perceived trustworthiness of masked and unmasked dominant faces did not change (*p*_FDR_ = .085, *d* = 0.18, 95% CI [0.01, 0.35]), masking did reduce the ability to judge the trustworthiness of submissive faces such that unmasked submissive faces were perceived as more trustworthy than masked submissive faces (*p*_FDR_ < .001, *d* = 0.43, 95% CI [0.26, 0.60]). This suggests that the cues covered by masks in submissive faces might be exclusively affecting perceptions of trustworthiness, while the cues that inform high facial dominance are not disrupted by masking. Follow-up comparisons further showed that the differentiation between dominant and submissive faces in terms of both judgments was statistically significant regardless of masking (see Table 2 and Fig. 3). These results support the hypothesis that the discrimination facial dominance is less disturbed by masking, regardless of judgment. Finally, the significant Face Dimension Pole × Judgment interaction, *F*(1, 69) = 118.95, *p* < .001, *η*_p_^2^ = .633, 95% CI [.494, .727], again indicates a negative relationship between judgments such that the higher the facial dominance the lower the perceived trustworthiness.

#### Trustworthiness versus dominance faces

A direct comparison between the effect sizes for the three-way interactions obtained for faces manipulated on trustworthiness (*η*_p_^2^ = .405, 95% CI [.233, .546]) and faces manipulated on dominance (*η*_p_^2^= .083, 95% CI [.003, .226]) faces suggests that the effect of masking on the discriminability of face poles in terms of trustworthiness and dominance impressions was stronger for CG faces varying in trustworthiness (CIs do not overlap). The overall pattern of results in Fig. 3 shows that masking consistently decreased the discriminability between face poles. And importantly, a comparison between masked and unmasked faces suggests that the discriminability between face poles was significantly reduced for both judgments, but only clearly so for faces manipulated in trustworthiness.

This pattern only partially aligns with our hypothesis that trustworthiness judgments would be more affected by masking relative to dominance judgments. It suggests that the way both traits are judged is more strongly disrupted by the presence of a mask when faces vary in trustworthiness relative to when they vary in dominance.

### Real-life faces

**Fig. 4:**
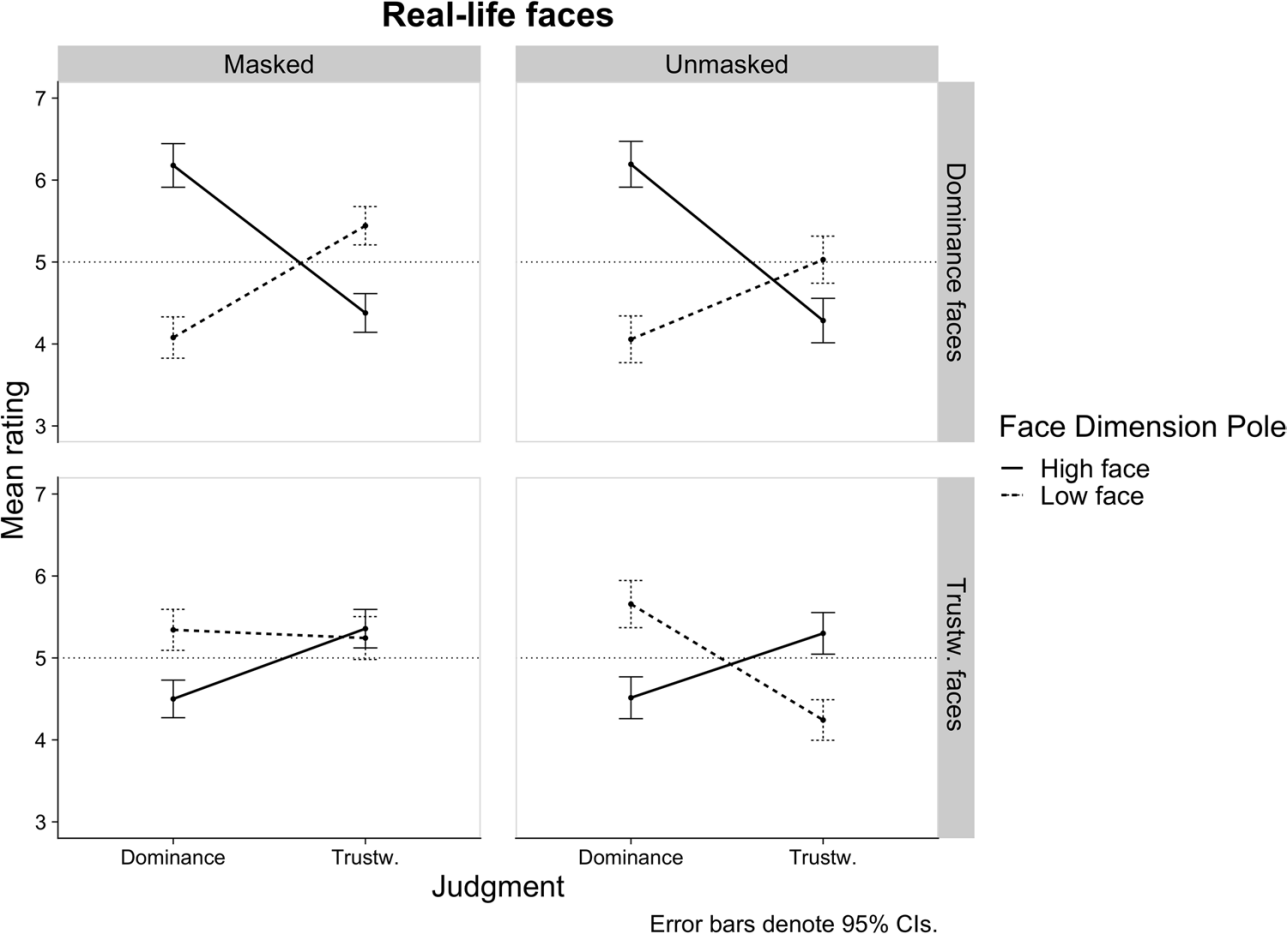
Study 1b plot of the three-way Judgment × Masking × Face Dimension Pole interactions by Face Dimension. Horizontal dotted line represents the middle point of the rating scale

The same analytical approach was applied to ratings of real-life faces. Figure 4 shows the pattern of the three-way interactions by Face Dimension for real-life stimuli. Table 3 lists all the *FDR*-corrected *p*-values and effect sizes for the differences between Face Dimension Pole levels for each condition of the three-way interactions.

**Table 3:** Study 1b: Effect sizes of the difference between the ratings of the high and low poles of a face dimension for each condition

			Face dimension pole			
Face dimension	Judgment	Masking	High *M* (*SD*)	Low *M* (*SD*)	High vs. Low	Cohen’s d [95% CI]
Dominance	Dominance	Masked	6.18 (1.56)	4.08 (1.49)	*p* < .001	1.13 [0.83, 1.44]
		Unmasked	6.19 (1.66)	4.06 (1.66)	*p* < .001	1.12 [0.82, 1.43]
	Trustw.	Masked	4.38 (1.46)	5.44 (1.47)	*p* < .001	-0.71 [-0.98, -0.45]
		Unmasked	4.29 (1.71)	5.03 (1.73)	*p* = .003	-0.38 [-0.63, -0.14]
Trustw.	Dominance	Masked	4.50 (1.38)	5.34 (1.49)	*p* < .001	-0.65 [-0.91, -0.39]
		Unmasked	4.51 (1.51)	5.66 (1.69)	*p* < .001	-0.73 [-1, -0.47]
	Trustw.	Masked	5.36 (1.51)	5.24 (1.62)	*p* = .611	0.08 [-0.15, 0.32]
		Unmasked	5.3 (1.54)	4.24 (1.55)	*p* < .001	0.69 [0.43, 0.96]

*n* = 140 for all cell means; positive (negative) Cohen’s *d* values indicate higher mean rating for the high (low) pole of face dimension. A Benjamini-Hochberg correction was applied to all *p*-values in this analysis

#### Trustworthiness faces

The three-way interaction was significant, *F*(1, 69) = 12.30, *p* < .001, *η*_p_^2^ = .151, 95% CI [.030, .308]. The simple interaction effects at each level of Judgment clarified that the Masking × Face Dimension Pole interaction was only significant for trustworthiness judgments, *F*(1, 69) = 20.92, *p* < .001, *η*_p_^2^= .233, 95% CI [.080, .391] (vs. Dominance judgments: *F*(1, 69) = 1.39, *p* = .242, *η*_p_^2^= .020, 95% CI [.000, .125]). Follow-up comparisons between the high and low face poles for each masking level, showed that, as expected, the discriminability between trustworthy and untrustworthy faces was drastically decreased by masking, but only in terms of perceived trustworthiness, to the extent that masked trustworthiness faces were no longer differentiated (see Table 3 and Fig. 4).

Comparisons between masked and unmasked faces of the same dimension and pole, deviated from the pattern obtained with CG faces. For real-life faces, masking only significantly affected trustworthiness impressions of untrustworthy faces, *p*_FDR_ < .001, *d* = -0.63, 95% CI [-0.87, -0.39] (vs. trustworthy faces: *t* < 1, *d* = -0.04, 95% CI [-0.27, 0.20]), such that masked untrustworthy faces were judged as more trustworthy. Such a result suggests an unforeseen beneficial effect of masking such that it increases the perceived trustworthiness of untrustworthy-looking faces. Regarding impressions of dominance, masking did not affect judgments of trustworthy faces, *t* < 1, *d* = 0.01, 95% CI [-0.22, 0.24], nor judgments of untrustworthy faces, *p*_FDR_ < .236, *d* = 0.20, 95% CI [-0.02, 0.43], suggesting low or no disruption of facial cues informing judgments of dominance in real-life faces varying in trustwortiness. Finally, the significant Face Dimension Pole × Judgment interaction, *F*(1, 69) = 61.66, *p* < .001, *η*_p_^2^ = .472, 95% CI [.303, .601], once again indicates a negative relationship between judgments.

#### Dominance judgments

As expected, we found no significant three-way interaction, *F* < 1, *η*_p_^2^ = .010, 95% CI [.000, .102], suggesting that masking did not significantly affect the discrimination between of face dimension and poles in terms of perceived trustworthiness and dominance. There were only three other significant effects: A Face Dimension Pole × Judgment interaction, *F*(1, 69) = 90.13, *p* < .001, *η*_p_^2^ = .566, 95% CI [.412, .676], again indicating a negative relationship between judgments. A main effect of Face Dimension Pole, *F*(1, 69) = 45.35, *p* < .001, *η*_p_^2^ = .397, 95% CI [.224, .539], clarifying that face dimension poles were differentiated as intended regardless of face dimension (see Table 3). And a main effect of Judgment dimension, *F*(1, 69) = 5.02, *p* = .028, *η*_p_^2^ = .068, 95% CI [.000, .206], showing that, overall, dominance judgments (*M* = 5.13 *SD* = 1.91) were higher than overall trustworthiness judgments (*M* = 4.78 *SD* = 1.66). No effects involving Masking achieved significance, suggesting that, as expected, dominance judgments were not affected by facial masking for faces manipulated on dominance.

#### Trustworthiness versus dominance faces

Relative to the findings with CG faces, the pattern of results for both judgments obtained with real-life faces offers support to our hypothesis that dominance impressions would be less affected by masking compared to trustworthiness impressions. This hypothesis is further substantiated by the pattern of effect sizes reflecting the differentiation between face poles in terms of perceived trustworthiness and dominance (see Fig. 4 and Table 3). In Fig. 4, the differences in discriminability emerge more clearly between masked and unmasked faces manipulated on trustworthiness when these are judged on trustworthiness. By contrast, the discriminability between face poles was not affected (or only negligibly so) when faces were judged on dominance, regardless of face dimension.

## Study 2

Study 1 suggests that occluding the bottom half of the face is more detrimental to the discrimination of facial trustworthiness than dominance. Our claim is that this effect is associated with the mere absence of information concealed by the mask. However, it is also possible that the effect was promoted by the observed act of the target being seen wearing a mask during a pandemic. In line with this alternative hypothesis, a recent study involving real-life faces showed that mask wearers were perceived as more trustworthy than they were when unmasked, and suggested the interpretation of this effect as promoted by a perceived compliance with an emerging social norm (Olivera-La Rosa et al., 2020). If such an effect is at play, perceived trustworthiness may be inflated for untrustworthy-looking masked faces. Consequently, this would mitigate the discriminability between high and low trustworthiness in masked targets. To confront these two hypotheses, Study 2 replicates the masked condition of Study 1 by replacing a face mask with a digital neutral square image occluding the bottom-half of a target face. In addition to the stimuli for which the top-half of the face was visible, we included stimuli for which only the bottom-half was visible, to serve as non-focal fillers in this study. These filler stimuli introduced variability in the face set, counteracted any potential strategy of exclusively focusing the attention on the upper face region throughout the rating task, and allowed additional exploratory analyses of facial impressions based on the bottom-face region.

We narrowed our focus exclusively to real-life faces, since the trait poles of these faces do not vary in gender – thus preventing the noise introduced by gender variation between the poles of the same stimulus. We expected to replicate the findings in Study 1 and observe a lower discriminability of facial trustworthiness compared to that of facial dominance, regardless of the type of object that conceals trait signal.

### Participants

Seventy people (50% female, 50% male, *M*_age_ = 27.3, *SD*_age_ = 5.62, age range = 18–40 years) were recruited via Prolific Academic to participate in the study in exchange for £0.75. Participants were selected using the same criteria applied in Study 1. The majority of our participants lived in the UK at the time of data collection (Ireland: 4.5%, UK: 92.5%, USA: 3%). All 70 participants were included in the analyses. Informed consent was obtained from all participants.

### Context of data collection

Data collection took place in the beginning of November 2020, during the second wave of the COVID-19 pandemic in the UK and most of Europe (House of Commons Library, 2021).

### Power considerations

Sample size was determined before any data analysis. Our sample size was established on the basis of available resources (maximum *N* = 70). A power simulation (similar to that of Study 1) suggested that a sample size of 70 participants would allow to detect a Face Dimension × Face Dimension Pole interaction with an effect size of *η*_p_^2^ = .38 with 100% power for dominance judgments, and *η*_p_^2^ = .10 with 73.65% power, for trustworthiness judgments. Note that this difference in power results from fine-tuning the simulation to expect lower effect sizes for differences in the perceived trustworthiness of face dimension poles (simulation report available at: https://osf.io/4xvem/).

### Stimuli

**Fig. 5:**
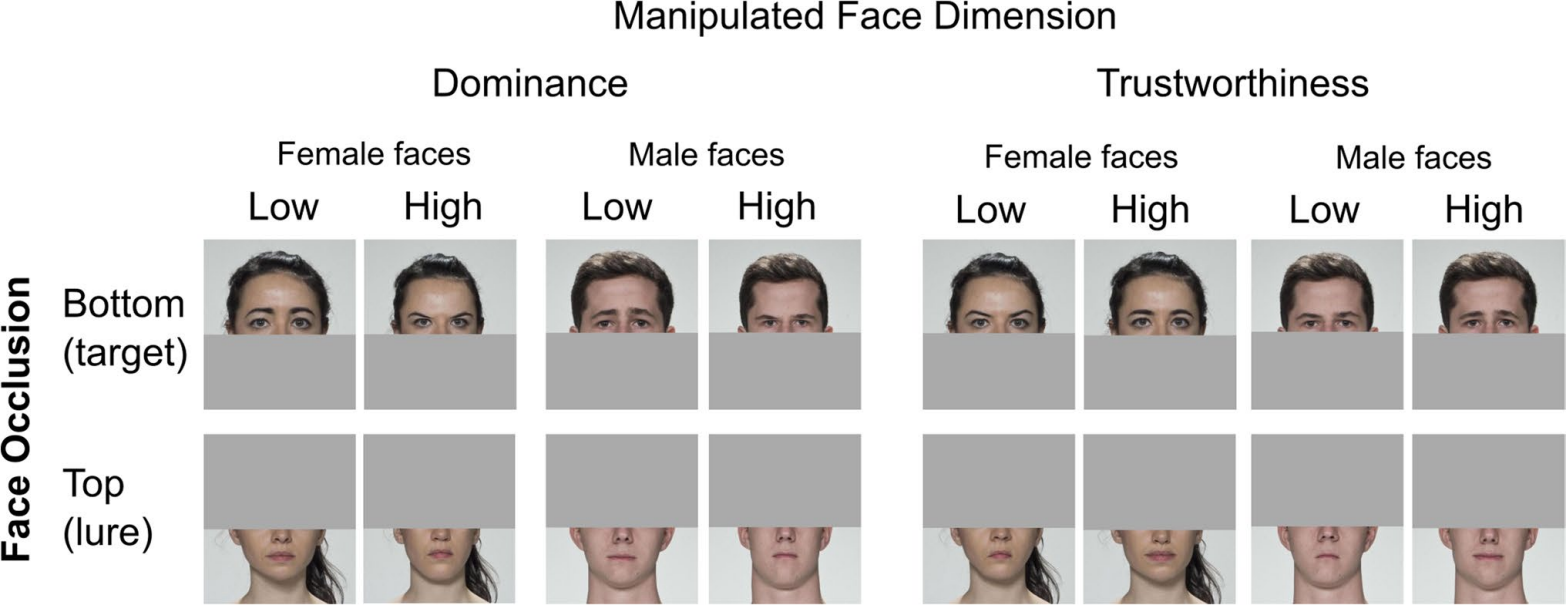
Exemplars of face stimuli used in Study 2. Grey square images occluding face regions automatically adjusted to facial shape using height of nose landmark as anchor. Original stimuli extracted from the materials generated by Oh et al. (2020)

Only real-life faces were used in Study 2. The stimulus set consisted of the same set used in Study 1 plus four additional face identities (two male, two female) randomly sampled from the previously unsampled identities in the original database (Oh et al., 2020). All faces were of White ethnicity and had a mean age of 27.2 years (*SD*_age_ = 5.51 years). For each face, we created a version where the top-half of the face was visible, and another where only the bottom-half was visible. To cover the top or bottom half of a face, we overlayed a grey square image onto the face picture, using the same central nose landmark previously used to mask faces as the point dividing the face picture in half. As in Study 1, this facial landmark was detected and adjusted to each stimulus using a similar programmatic approach which additionally produced the grey images. Exemplars of the stimuli are shown in Fig. 5.

### Procedure

The procedure was in every way identical to that of Study 1 with some exceptions. The instructions were adapted to the new stimulus set and informed participants that they would see “different exemplars of human faces partially covered by a grey wall” that would “either cover the top of the face or the bottom of the face.” Both types of occluded faces were presented within the same block in randomized order. After completing the task, participants provided their demographical information and rated how easy it was to use each judgment scale on a 9-point scale ranging from 1 (Very difficult) to 9 (Very easy). Next, they completed additional tasks involving face ratings collected for exploratory purposes that fall outside the scope of the current paper. Finally, they were thanked, debriefed, and compensated.

### Results and discussion

#### Inter-rater agreement

Overall, there was high agreement in participants’ ratings (see Table 1). The lowest agreement was observed for dominance judgments of trustworthiness faces, and the highest agreement for dominance judgments of dominance faces.

#### Difficulty of using judgment scales

Again, the dominance scale was perceived as slightly easier to use (*M* = 4.86, *SD* = 2.31) compared to the trustworthiness scale (*M* = 4.53, *SD* = 2.27). However, this difference was not significant, *t*(69) = 1.14, *p* = .26, *d* = 0.14.

#### Statistical analyses

We followed a similar analytical approach to that in Study 1 with the exception that the ANOVA was estimated via mixed-effects modelling to prevent any information loss triggered by imbalanced observations in the design (see Table 4). Specifically, our mixed model specified face dimension, face dimension pole, and judgment as fixed effects, and allowed the intercepts and slopes of all fixed effects to vary across participant. This model is comparable to a 2 (Face Dimension: dominance vs. trustworthiness) × 2 (Face Dimension Pole: high vs. low) × 2 (Judgment: dominance vs. trustworthiness) within-participants ANOVA with judgment ratings as the dependent variable. Reported degrees of freedom were obtained using Satterthwaite’s approximation method (Kuznetsova et al., 2017; Singmann et al., 2021). We report results for judgments of faces with the bottom-half occluded by a square image. To clarify, in our analyses we categorized the face stimuli into target (bottom half occluded) and distractor/non-focal faces (top half occluded). Our focus was on the target faces as they mimicked the masked faces in Study 1, but without face masks. The remaining non-focal faces were discarded from the main analyses, as we had not prespecified hypotheses for them. (These were, however, informative for exploratory analyses.)

**Table 4:** Study 2: Effect sizes of the difference between the ratings of the high and low poles of a face dimension for each judgment dimension

Face dimension	Judgment	Face pole	*M* (*SD*)	High vs. Low	Cohen’s d [95% CI]	n
Dominance	Dominance	High	6.10 (1.83)	*p* < .001	1.43 [1.06, 1.80]	240
		Low	4.58 (1.63)			180
	Trustw.	High	3.99 (1.64)	*p* < .001	-1.48 [-1.85, -1.10]	210
		Low	5.53 (1.53)			210
Trustw.	Dominance	High	4.97 (1.61)	*p* = .020	-0.59 [-0.99, -0.19]	210
		Low	5.43 (1.64)			210
	Trustw.	High	5.35 (1.53)	*p* = .108	0.30 [-0.09, 0.69]	240
		Low	5.01 (1.50)			180

Positive (negative) Cohen’s *d* values indicate higher mean rating for the high (low) pole of face dimension. A Benjamini-Hochberg correction was applied to all *p*-values in this analysis

The three-way interaction^1^ was significant, *F*(1, 75.94) = 97.89, *p* <.001, *η*_p_^2^ = .563, 95% CI [.416, .669], suggesting that the effect of masking on the discriminability of face poles differed between trustworthiness and dominance faces. Analyses of the simple interactions effects by Face Dimension are described below. There was also a significant Face Dimension Pole × Judgment interaction, *F*(1, 91.97) = 31.21, *p* <.001, *η*_p_^2^ = .253, 95% CI [.115, .392], once again indicating a negative relationship between the two judgments.

##### Trustworthiness faces

The Face Dimension Pole × Judgment interaction was significant, *F*(1, 265.71) = 12.14, *p* < .001, *η*_p_^2^ = .091, 95% CI [.058, .130], indicating that the ability to discriminate between trustworthy and untrustworthy faces was more strongly reduced for judgments of trustworthiness, *F*(1, 61.52) = 12.14, *p* =.072, *η*_p_^2^= .052, 95% CI [.000, .191], than for judgments of dominance, *F*(1, 65.54) = 9.32, *p* = .003, *η*_p_^2^= .124, 95% CI [.015, .282] (means are shown in Table 4). This replicates the effect obtained in Study 1b for trustworthiness judgments of masked real-life faces.

##### Dominance faces

The Face Dimension Pole × Judgment interaction was significant, *F*(1, 69.58) = 105.18, *p* < .001, *η*_p_^2^= .602, 95% CI [.456, .703], indicating that participants were equally able to discriminate between dominance and submissive faces in terms of perceived trustworthiness, *F*(1, 69) = 71.68, *p* < .001, *η*_p_^2^= .510, 95% CI [.345, .631], and perceived dominance, *F*(1, 69) = 63.88, *p* < .001, *η*_p_^2^= .481, 95% CI [.313, .608] (see Table 4). This suggests that the occlusion of the bottom-half of faces varying in dominance does not significantly disrupt how they are judged in terms of dominance or trustworthiness.

##### Trustworthiness versus dominance faces

**Fig. 6:**
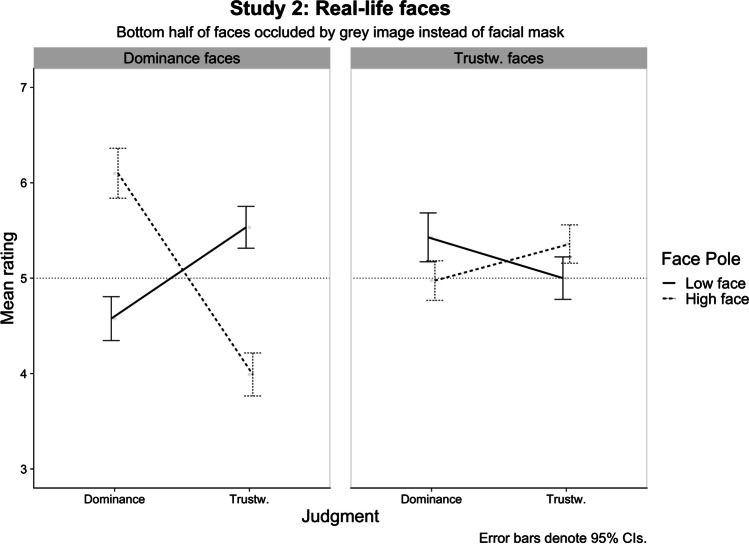
Study 2 plot of the three-way Face Dimension × Face Dimension Pole × Judgment interaction. Horizontal dotted line represents the middle point of the rating scale

The overall pattern of results illustrated in Fig. 6 shows that the occlusion of the bottom-half of the face had a stronger impact on judgments of faces manipulated to vary in trustworthiness than on faces manipulated on dominance. And importantly, it clarifies that the mere removal of facial information is sufficient to reduce the discriminability of trustworthiness in a face. Hence, an explanation of this effect based on perceptual processes is more likely than one based on social norms conveyed by face masks.

##### Non-focal stimuli

Additional exploratory analyses of the effect sizes obtained with non-focal stimuli (top half occluded) further indicated that the discriminability between the perceived trustworthiness of trustworthy and untrustworthy faces was higher when only their bottom halves were visible, *d* = 1.08, 95% CI [0.68, 1.47], compared to when they were occluded, *d* = 0.30, 95% CI [-0.09, 0.69]. And that the discriminability between the perceived dominance of dominant and submissive faces was lower when only their bottom halves were visible, *d* = 0.67, 95% CI [0.33, 1.01], compared to when they were occluded, *d* = 1.43, 95% CI [1.06, 1.80] (analyses available at: https://osf.io/4xvem/).

## General discussion

The current work investigated the impact of occlusions of the bottom half of the face on facial impressions of trustworthiness and dominance. Across the two studies, results show that this type of occlusion is more detrimental for the perception of trustworthiness than for the perception of dominance.

Both Study 1a and Study 1b showed that masking a face clearly affected how participants’ judgments correctly discriminated between trustworthy and untrustworthy faces and that the same did not occur for faces manipulated in dominance. Regardless of masking, participants discriminated well between dominant and submissive faces. This pattern occurred with both artificial (Study 1a) and real-life faces (Study 1b). Study 2 clarified that the asymmetry of the impact of the mask on trustworthiness and dominance impressions is more likely to be the result of the mere occlusion of facial information, rather than the result of any attributes that are exclusive to a face mask.

Regardless of the method by which the bottom-face region was occluded, participants discriminated between trustworthy and untrustworthy faces. This suggests that some facial cues to trustworthiness remain visible when the bottom-face region is masked and convey sufficient signal to allow for a discrimination between facial trustworthiness poles. Indeed, this has been suggested by previous studies. Besides the mouth region, the eyes region and its involvement in smiling expressions are also relevant for trustworthiness inferences (Krumhuber et al., 2007; Oosterhof & Todorov, 2009).

The present studies consistently suggest that trustworthiness and dominance judgments are not entirely independent. In agreement with previous work (e.g., Dotsch & Todorov, 2012; Oliveira et al., 2020; Oosterhof & Todorov, 2008), the overall association between these two dependent variables was negative. This negative association between judgments could have resulted in a process whereby facial dominance was discriminated by trustworthiness judgments and facial trustworthiness by dominance judgments. This could be an alternative explanation to the one presented above regarding why a masked face may convey sufficient signal to allow for a discrimination between facial trustworthiness poles: perceivers may have just been supported by dominance inferences. Alternatively, it is also possible that these inferences result mainly from a more general valence evaluation, instead of specific inferences on either of the two dimensions. If so, one could additionally take into account previous findings showing a higher overlap of trustworthiness judgments with general valence in social face evaluation (e.g., Oh et al., 2020; Oliveira et al., 2020; Oosterhof & Todorov, 2008).

In sum, the current data are clearly suggesting that the concealment of the bottom-face region by means of an opaque object (e.g.*,* a face mask, but not exclusively so) interferes to a greater extent with facial impressions of trustworthiness than with facial impressions of dominance, and that the features located in the top-face region are either directly or indirectly conveying information about trustworthiness. Furthermore, these findings extend and align with recent findings showing that facial judgments of trustworthiness are more strongly disrupted by face masks than judgments of dominance, for both White and Black faces (Oldmeadow & Koch, 2021).

### Differences between artificial and natural faces

Although masks affected the discriminability of facial trustworthiness, their impact was less pronounced in judgments of artificial faces. There may be different reasons underlying why the relevance of top-face facial cues for trustworthiness judgments was less evident in real-life stimuli. Our real-life stimuli may be expressing less variability on the cues that participants relied upon to discriminate facial trustworthiness while they were judging artificial stimuli. Unlike real-life faces, the specific set of artificial faces used in our studies offered additional cues to judgments, since they capture the natural confound between gender stereotypes and social traits (Todorov et al., 2013b), and variability in skin reflectance. For example, the high pole of an artificial face manipulated in dominance looks masculine, while its low pole counterpart for the same face identity (or model to be precise) looks feminine. By contrast, gender cues remain invariant across the poles of every real-life stimulus. These additional cues to gender in artificial faces introduced noise in the observed judgments, making it unclear whether gender stereotypes could be filling in the gaps of missing facial information in judgments of the two poles of the same face identity/model. Another possibility could be the activation of another process, whereby the perception of the “trustworthy” behavior of wearing a mask would mitigate perceptions of untrustworthiness (thereby reducing the discriminability between poles) (see Olivera-La Rosa et al., 2020). However, Study 2 clarified that the decreased discriminability of trustworthiness in faces could be parsimoniously explained by the mere absence of facial information.

### Limitations

There are several limitations that should be considered while interpreting the current results. Our stimulus sets were entirely composed of White faces (see Cook & Over, 2021). Although this helped us to reduce the complexity of our study designs, it does restrict the generalizability of these effects to the full range of facial ethnicities (but see Oldmeadow & Koch, 2021, for similar work incorporating face ethnicity). Second, we did not collect data on attitudes towards face mask wearing or how much familiarity (or expertise) our participants had with them. Future studies could examine the moderating effects of such variables on perceptions of masked faces.

Another limitation is that the high and low poles of the real-life stimuli used in our studies are somewhat exaggerated, which could have mitigated their realism contrary to our intention of increasing it. At the same time, such extreme exemplars could have acted as a more severe test to our hypothesis that the poles of facial trustworthiness would be harder to discriminate, since the caricaturized faces are amplifying the difference between poles. Future studies could clarify this possibility by creating continua of facial trustworthiness and dominance for each face identity to investigate how using face poles that are symmetrically and decreasingly distant from the mid-face of a continuum would impact the ability to discriminate them in trustworthiness and dominance.

### Practical implications

The relevance of these findings becomes especially clear in the social context of a pandemic. The widespread use of face masks could be creating a demanding social environment where not only it is harder to recognize a person (Freud et al., 2020), but also harder to rely on the processes by which we infer trustworthiness. These additional implications should be further investigated. One possibility is that the efficiency of social judgments will remain undisturbed, given the evidence suggesting that people can easily extract information to support accurate social judgments via “thin slices” of behavior (Ambady, 2010; Ambady et al., 2000; Ambady & Rosenthal, 1993; Kenny et al., 1992). Alternatively, if the activation of more cognitive resources is required to prevent biases, more cognitive depletion should be expected in our social interactions (e.g., Gilbert et al., 1988). By itself, this is a source of stress with further implications on other cognitive processes that would favour other types of biases (e.g., stereotyping; Carter et al., 2015; Macrae & Bodenhausen, 2001). This possibility seems to be endorsed by the current data. The discrimination of facial trustworthiness in masked artificial faces could have been aided by the presence of cues reflecting gender-trait associations in these stimuli, as a higher reliance on stereotypical information might have filled in for the missing facial information. Recent findings by Oldmeadow and Koch (2021) showing that face masks reduce the impact of race effects on facial impressions might be informative with regard to such a possibility, although it remains unclear whether the same reduction would occur for facial cues to gender. Finally, it might be worthwhile to extend the present investigation to other forms and consequences of face occlusion occurring in less ephemeral contexts than pandemics, such as those involving religious norms (e.g., niqab, which only leaves the eyes region exposed) or highly polluted environments (e.g., smog).

## Data Availability

The anonymized data, face stimuli, study instructions, and code to reproduce the data analyses and results reported in the article are publicly available at the Open Science Framework repository: https://osf.io/4xvem/

## Footnotes

1 A marginally significant effect of scale positioning was observed indicating a very weak interaction of scale positioning with the Face Dimension × Face Pole × Judgment effect, *F*(1, 75.57) = 4.13, *p* = .046. Follow-up analyses clarified that the difference was circumscribed to judgments of dominance faces. However, the pattern of effects for these faces and their significance was the same for both orders of scale positioning. Therefore, we conclude that this marginal effect did not have any meaningful impact on our main results. This analysis is available in the Online Supplementary Materials.
